# Ontario Veterinary Medical Society

**Published:** 1887-01

**Authors:** 


### ONTARIO VETERINARY MEDICAL SOCIETY.

For a number of years a Veterinary Medical Society has been in existence in connection with the college. Through the energy of the president and the enthusiasm of the students, this Society has been exceedingly successful. The meetings are held twice a week, the attendance at each meeting ranging from two hundred and fifty to three hundred. Two or more essays or communications are read each evening by the members, and each essayist is expected to defend his paper against the criticism of any one of the two or three hundred fellow-students who form his audience. The training in public speaking and debating received in this Society proves of value in after life to those taking part in it.

At the regular meeting of this Society, an interesting pathological specimen was presented ; this being the heart and aorta from a patient who had suffered from complete aphonia during the later months of his life. So puzzling was the ante-mortem examination that there was wide diversity of opinion between the experts in regard to the condition present. Post-mortem, this was found to be an aneurism of the aorta, pressing upon the left recurrent laryngeal nerve. Paralysis of this nerve followed, with complete loss of voice. In view of this occult pathology of "roaring" in the horse, such specimens may be of great importance. In the many cases of the above affection coming under the notice of veterinarians, the roaring has generally to be credited to atrophy and degeneration of muscles of the left side of the larynx. The cause of the atrophy is generally wrapped in obscurity. Very seldom does a post-mortem examination reveal aneurism, or tumor of any kind, pressing upon the left recurrent nerve. So difficult is it to find a cause that Professor Williams holds this condition to be one of "Paralysis Atrophica" (Progressive Muscular Atrophy), in which, he says, the the paralysis begins in the muscles themselves. This is a suggestion worthy of careful investigation, although the supposition that this form of paralysis originates in the muscles themselves has been disproved by more recent investigation. "Progressive muscular atrophy originates from functional or structural changes in the nervous centers or their nerves" (J. Lockhart Clarke). Even granting that laryngeal paralysis in the horse is due to progressive muscular atrophy, the above remark would cause investigation again to be directed to the nerves, to discover the cause. Unfortunately for comparative pathology, no examination, in the case above spoken of, was made of either the laryngeal muscles or of the recurrent nerve in its structure. But in this patient the aphonia was complete ; in cases of roaring the loss of voice is not necessarily so.

